# Human endometrial stem cells confer enhanced myocardial salvage and regeneration by paracrine mechanisms

**DOI:** 10.1111/jcmm.12100

**Published:** 2013-07-09

**Authors:** Zhi Jiang, Xinyang Hu, Hong Yu, Yinchuan Xu, Lihan Wang, Han Chen, Huiqiang Chen, Rongrong Wu, Zhaocai Zhang, Chunsheng Xiang, Keith A Webster, Jian-an Wang

**Affiliations:** a Department of Cardiology, Second Affiliated Hospital, Zhejiang University School of MedicineHangzhou, China; b Key lab of cardiovascular disease, Second Affiliated Hospital, Zhejiang University School of MedicineHangzhou, China; c Zhejiang-California International NanoSystems Institute Zhejiang UniversityHangzhou, China; d Vascular Biology Institute, Miller School of Medicine, University of MiamiMiami, FL, USA

**Keywords:** endometrial stem cells, myocardial infarction, paracrine, regeneration, apoptosis, angiogenesis

## Abstract

Human endometrial stem cells (EnSCs) have the potential to be ‘off the shelf’ clinical reagents for the treatment of heart failure. Here, using an immunocompetent rat model of myocardial infarction (MI), we provide evidence that the functional benefits of EnSC transplantation are principally and possibly exclusively through a paracrine effect. Human EnSCs were delivered by intramyocardial injection into rats 30 min. after coronary ligation. EnSC therapy significantly preserved viable myocardium in the infarct zone and improved cardiac function at 28 days. Despite increased viable myocardium and vascular density, there was scant evidence of differentiation of EnSCs into any cardiovascular cell type. Cultured human EnSCs expressed a distinctive profile of cytokines that enhanced the survival, proliferation and function of endothelial cells *in vitro*. When injected into the peri-infarct zone, human EnSCs activated AKT, ERK1/2 and STAT3 and inhibited the p38 signalling pathway. EnSC therapy decreased apoptosis and promoted cell proliferation and c-kit+ cell recruitment *in vivo*. Myocardial protection and enhanced post-infarction regeneration by EnSCs is mediated primarily by paracrine effects conferred by secreted cytokines that activate survival pathways and recruit endogenous progenitor stem cells. Menstrual blood provides a potentially limitless source of biologically competent ‘off the shelf’ EnSCs for allogeneic myocardial regenerative medicine.

## Introduction

Myocardial infarction (MI) is a major cause of morbidity and mortality worldwide [1]. Stem cell therapy has the potential to promote myocardial regeneration [2]. Autologous and allogeneic mesenchymal stem cells (MSCs) have been tested clinically, and most of the trials to date have reported safety but modest efficacy [3]. It has been proposed that the generation of ‘off the shelf’ allogeneic stem cells or their products may overcome the limitations of autologous cell therapy and improve the clinical outcome [4]. Endometrial stem cells (EnSCs) with MSC characteristics are isolated from menstrual blood [5, 6]. A unique feature of these cells is that they can be easily obtained by non-invasive procedures from healthy young women. The MSC characteristics of EnSCs also guarantee immunoprivilege in allogeneic hosts. EnSC may be an ideal cell type to generate ‘off the shelf’ cell products [7, 8].

Most preclinical studies have shown that transplanted stem cells show low rates of engraftment and limited differentiation [9, 10]. Rather it appears that functional benefits of stem cell therapy for the heart are mediated principally by paracrine effects [11]. Therefore, the composition and quantity of secreted cytokines is of paramount importance for therapeutic efficacy and this is cell-type dependent [12]. In this study, we characterized the profile of cytokines secreted from human EnSCs and analysed the associated paracrine properties *in vitro* and *in vivo*. Using an immunocompetent rat model, we show that direct injection of EnSCs into the peri-infarct zone immediately following coronary artery occlusion reduced apoptosis, activated survival kinases, stimulated endogenous regeneration, preserved viable myocardium within the area of ischaemia and improved myocardial function. The study demonstrates that human EnSCs are a rich source of protective cytokines and suggests that EnSC therapy for MI is primarily by paracrine pathways.

## Materials and methods

Detailed experimental protocols are described in the supplemental information.

### Cell sources

Human BMMSCs from three donors were purchased from Cyagen Bioscience (Shanghai, China). Human umbilical vein endothelial cells (HUVECs) were purchased from AllCells (Shanghai, China). Human EnSCs from three donors were provided by S-Evans Biosciences (Hangzhou, China). All human cells were used under the approval of the Clinical Ethics Committee of Zhejiang Province Medical Institute, and conform to the declaration of Helsinki.

### Flow cytometric analysis

Endometrial stem cells were suspended in 100 μl PBS at 5 × 10^5^ per tube and incubated with primary antibodies or isotype-matched control antibodies on ice for 1 hr. Cells were washed with PBS and analysed by flow cytometry (Becton Dickinson, Franklin Lakes, NJ, USA).

### Collection of conditioned medium

Endometrial stem cells were grown to 90% confluence and the culture medium replaced with 3 ml of DMEM containing 2% foetal bovine serum. After 24 hrs of normoxia incubation, the conditioned medium was collected and used for angiogenesis assays.

### Tube formation assay

Human umbilical vein endothelial cells were seeded into Matrigel prepared wells at 8 × 10^4^ cells per well with EnSCs conditioned medium or control DMEM containing 2% foetal bovine serum. Images were acquired by phase-contrast microscopy (Leika, Wetzlar, Germany).

### ELISA

8 × 10^5^ EnSCs or BMMSCs were seeded into 25 cm^2^ flasks, incubated for 12 hrs and subjected to normoxia or hypoxia for 24 hrs. Supernatants were collected and TGF-β2, EGF and VEGF quantified by Quantikine ELISA kit (R&D, Minneapolis, MN, USA), following the manufacture's instruction.

### Isolation of neonatal rat ventricular cardiomyocyte

Primary cultures of neonatal rat ventricular cardiomyocytes were prepared as described previously [13]. Briefly, hearts from 2-day-old Sprague-Dawley rats were minced and dissociated with 0.1% trypsin (Gibco, Grand Island, NY, USA). Cardiomyocytes were purified by differential adhesion to plastic on 24- or 6-well plates (Corning, Tewksbury, MA, USA).

### Coculture assay

Coculture experiments were performed in 24-insert transwell systems (Corning, 3 μm pore size). EnSCs were seeded on the semi-permeable membranes of the inserts and incubated for 24 hrs to allow adherence. Inserts with adherent EnSCs were incubated with cardiomyocyte cultures. Inserts without EnSCs served as controls.

### Rat model of MI and EnSCs implantation

The study design is illustrated in Figure S1. All experiments were approved by the Animal Care and Use Committee of Zhejiang Province Medical Institute and meet the standard of the U.S. National Institutes of Health. MI by permanent coronary artery ligation was implemented on Sprague-Dawley rats (200–250 g) as described previously [14]. Briefly, rats were intubated and ventilated under general anaesthesia with pentobarbital (50 mg/kg of bodyweight). MI was induced by ligation of the left anterior descending coronary artery with a 6-0 silk suture. Sham-operated group received the same surgery without coronary ligation. Thirty minutes after LAD ligation, 1.5 × 10^6^ EnSCs labelled with DiI (Invitrogen, Grand Island, NY, USA) in a final volume of 150 μl were injected into the ischaemic border zone at five sites. Control rats received the same volume of PBS without cells.

### Echocardiography

Echocardiography was performed at baseline, 7 and 28 days after MI. Rats were anesthetized with pentobarbital (50 mg/kg of bodyweight). Trans-thoracic two-dimensional and M-mode echocardiographic images were collected at the papillary muscle level using a Vevo 2100 (VisualSonics, Toronto, Ontorio, Canada) by a blinded investigator.

### Positron emission tomography

Rats were injected with 0.6 mCi 18-FDG by tail vein. Thirty minutes later, the animals were anesthetized by inhalation of 2% isoflurane and placed in a spread prone position on a dedicated holder for imaging. The region of interest (ROI) method was used to measure the standard uptake volume (SUV) of infarct regions (infarction SUV) and normal regions (normal SUV) [15]. The relative viability index was used to evaluate the relative volume of viable myocardium in the infarcted regions.

### Histology

Rats were killed at 2, 7 and 28 days after MI. The hearts were quickly excised and embedded in optimal cutting temperature compound (Sakura Finetek, Torrance, CA, USA). Frozen sections of LV samples were cut at 7-μm thickness and stored at −80°C. Antibodies and the methodology of immunofluorescent staining are described in Data S1. Briefly, the infarct zone was defined by collagen type I staining and the adjacent endocardial and pericardial zone by Troponin I staining, excluding intact myocardium in the infarct border. The Troponin I positive zone in the infarct zone was defined as viable myocardium. The area of infarction and viable myocardium were quantified using Image-Pro Plus. Myocardial fraction was determined by [myocardium area/infarct area]×100%. Infarct size was calculated by dividing the sum of the endocardial and epicardial lengths of the infarct zone by the sum of the total epicardial and endocardial circumferences of the LV.

### Terminal deoxynucleotidyl transferase biotin-dUPT nick end labelling (TUNEL)

TUNEL staining (Promega, Madison, WI, USA) was used to visualize cell death of cultured cells and tissue sections following manufacture's instruction.

### Statistical analysis

Student's *t*-tests were used to analyse differences between two groups. One-way anova tests were used to analyse differences when three or more groups were compared. Data are presented as mean ± SEM. Changes were considered statistically significant if the *p* value was less than 0.05.

## Results

### Characterization of EnSCs

**Fig. 1:**
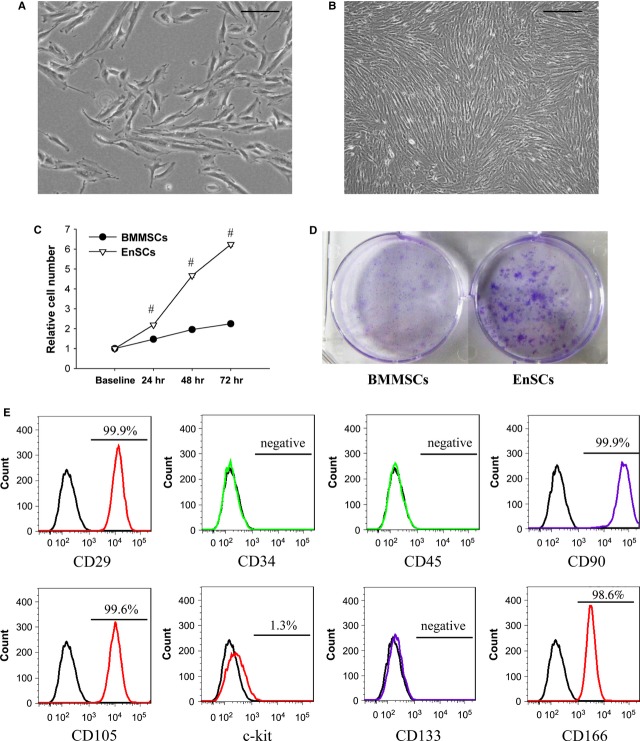
Morphology and phenotype of human EnSCs. (**A**) Phase-contrast microscopic view of normal cultured 30% confluent EnSCs, passage 9. Scale bar denotes 100 μm. (**B**) Phase-contrast microscopic view of normal cultured 100% confluent EnSCs, passage 9. Scale bar denotes 200 μm. (**C**) Quantification of relative cell number. Cell number measured at 24, 48 and 72 was standardized by baseline. #*P* < 0.01 *versus* BMMSCs. (**D**) Representative pictures of colony formation assay. (**E**) Flow cytometric analysis of cell surface marker on EnSCs. EnSCs showed mesenchymal characteristics.

Consistent with previous studies, EnSCs appeared as adherent spindle-like cells with round and centrally located nuclei (Fig. 1A) that formed swirled structures in monolayers (Fig. 1B). The proliferation rate of EnSCs was approximately threefold greater than that of BMMSCs (Fig. 1C). EnSCs also performed significantly better in colony assays as compared with BMMSCs (Fig. 1D). Flow cytometric analysis of surface markers (Fig. 1E) showed that the EnSCs were negative for CD34, CD45 and CD133, which are specific for hematopoietic cells and endothelial progenitors, and close to 100% positive for CD29, CD90, CD105 and CD166, which are considered to be specific for MSCs. C-kit expression was low in EnSCs.

### EnSCs transplantation improved cardiac function

**Fig. 2:**
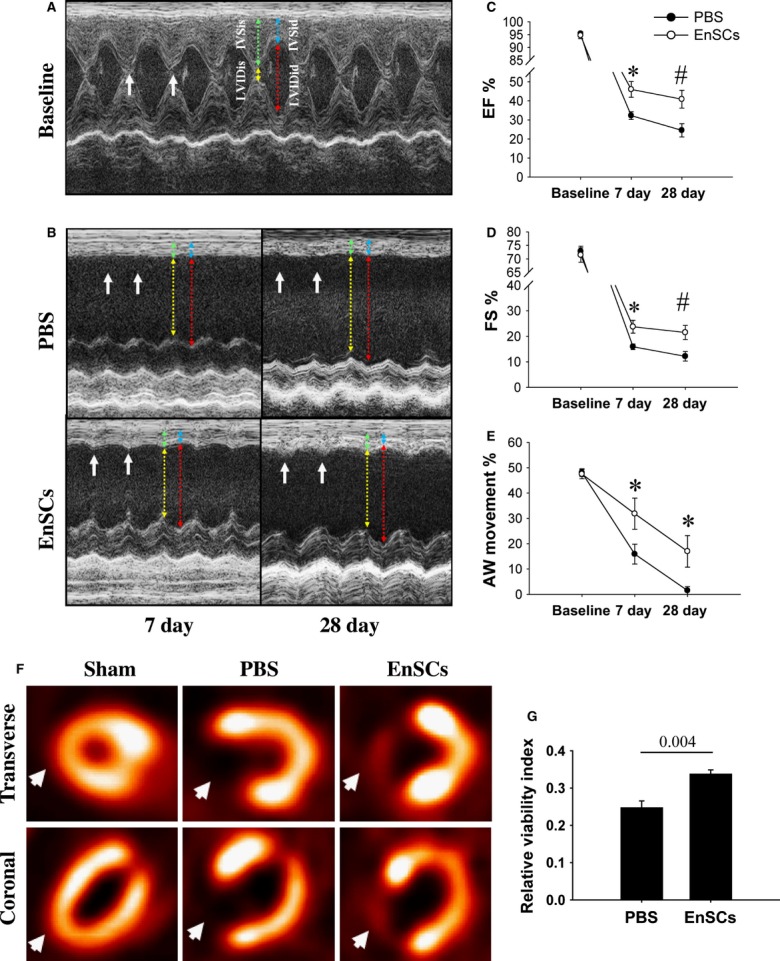
Functional benefits after the transplantation of EnSCs. (**A** and **B**) Representative M-mode echocardiographic images. The anterior wall (AW) movement was slightly preserved in EnSC group. Coloured lines showed the measurement of AW thickness and LV diameter. (**C** and **E**) Quantitative analysis of echocardiography (*n* = 8–14/group at each time-point). EnSCs transplantation increased ejection fraction, fractional shortening and improved AW movement at both 7 and 28 days. *P < 0.05 *versus* PBS group; #*P* < 0.01 *versus* PBS group. (**F**) Representative microPET images at transverse and coronal section at 28 days. PBS group showed radioactive defects as compared with sham group, but EnSC group showed low and distinct radioactivity in the infarct zone (white arrows), indicating viable cardiomyocytes in the zone. (**G**) Quantification of viable myocardium. The relative viability index was calculated by the region of interest method (*n* = 4/group). P values are shown at the top of bars.

We first validated the functional benefits of EnSCs transplantation in a rat MI model of permanent coronary occlusion. EnSCs were delivered by intramyocardial injection into the infarct border 30 min. after coronary ligation. Cardiac function was evaluated by echocardiography (Fig. 2A and B). Ejection fraction (EF), fractional shortening and anterior wall (AW) movements were each better preserved in the EnSC group as compared with the PBS group at 7 days after MI, and this effect was sustained at 28 days (Fig. 2C–E). The improved AW movement is indicative of functional myocardium in the infarct zone. To further assess the viable myocardium, 18-FDG microPET scans of the heart were performed at 28 days. No radioactive signal was detected in the infarct scar of the PBS group, whereas the EnSC group showed low but distinct radioactivity in the infarct zone (Fig. 2F), indicating viable myocardium within the infarction. The relative viability index was higher in the EnSC group compared with the PBS group (Fig. 2G). The results suggest that transplantation of EnSCs conferred preserved functional myocardium after MI.

### EnSC transplantation increased myocardium volume without significant differentiation

**Fig. 3:**
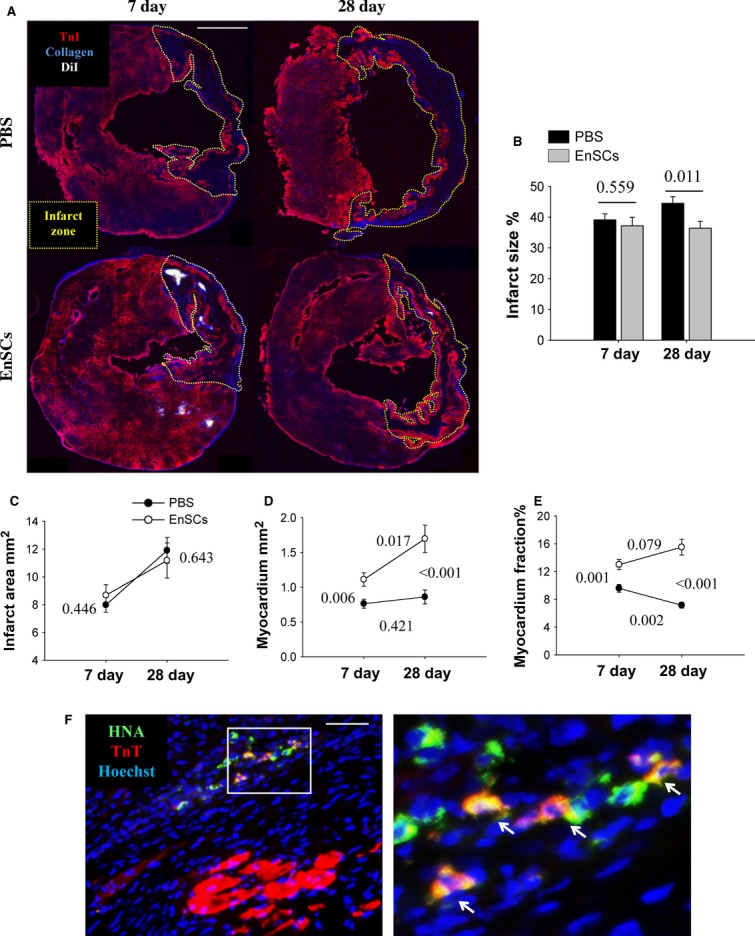
Transplantation of EnSCs preserved myocardium and enhanced myocardium regeneration, but few directly differentiated into cardiomyocytes *in vivo*. (**A**) Representative double staining of TnT in red and collagen type I in blue to show myocardium and scar at transverse section. Yellow dotted box labelled the infarct zone. Scale bar denotes 2 mm. (**B** and **C**) Quantification of infarct size and infarct area (*n* = 9–11/group at each time-point). The infarct size did not differ at 7 days, but was larger in PBS group than EnSC group at 28 days. P values are shown in the figures. (**D** and **E**) Quantification of myocardium area and myocardium fraction (*n* = 9–11/group at each time-point). The EnSC group had larger myocardium area and higher myocardium fraction than PBS group. The increased myocardial area in EnSC group from 7 to 28 days indicated a small proportion of myocardial regeneration. P values were shown in the figures. (**F**) Representative images of human nuclear antigen–positive and TnT-positive cells at 28 days (white arrows). Scale bar denotes 50 μm.

Double immunofluorescent staining of Troponin T (TnT) and collagen type I was used to measure viable myocardium in the infarct zone (Fig. 3A). There was no difference in infarct size between groups at 7 days (Fig. 3B), indicating a similar area at risk in all hearts by coronary ligation. The myocardial area and fraction were larger in the EnSC group at 7 days as compared with the PBS group (Fig. 3D and E), consistent with a protective effect of EnSCs on the infarcted myocardium. The myocardial area increased in the EnSCs, but not PBS group from 7 to 28 days (Fig. 3D). In contrast, the myocardial fraction did not change in EnSC group, but declined from 7 to 28 days in PBS group (Fig. 3E). Because the infarct area was similar at 28 days (Fig. 3C), the results suggest endogenous myocardial regeneration in the EnSCs transplantation group.

To address the mechanism of myocardial preservation, immunostaining of the recovered heart tissue was performed to identify possible differentiation of EnSCs into cardiomyocytes. Human EnSCs were detected by a human nuclear antigen (HNA)-specific antibody. Very few HNA-positive cells (273) were found at 28 days and only 9 (3.2%) of these apparent EnSCs expressed TnT (Fig. 3F). The morphology of the HNA+/TnT+ cells was non-striated; they appeared to be smaller than host cardiomyocytes and were separated physically from the host striated muscle suggesting that they were not physically integrated into host sarcomeres. Because both survival and differentiation of transplanted cells were very low, we conclude that differentiation does not contribute significantly to myocardial regeneration or preservation.

### Paracrine effect of EnSCs *in vitro*

Paracrine actions have been reported to play key roles in stem cell therapy for myocardial repair [11]. To test for this in our model, we profiled cytokines in EnSCs cultured under normoxia or simulated ischaemia (hypoxia/serum deprivation). We found that the cytokine mRNA expression profile of EnSCs differed markedly from that of BMMSCs (Figure S2A). EnSCs expressed higher level of EGF, periostin, Ang1 and PDGF compared with BM-MSCs under both culture conditions. ELISA was performed to quantify secreted cytokines. In these assays, we observed significantly higher levels of TGFβ2, EGF and nitric oxide (NO) secreted from EnSCs as compared with BM-MSCs under both culture conditions (Figure S2B). EnSCs did not secret VEGF under normoxia, but VEGF secretion was induced by simulated ischaemia. These results demonstrate that EnSCs secrete a distinct set of cytokine and growth factors that could be important therapeutically.

**Fig. 4:**
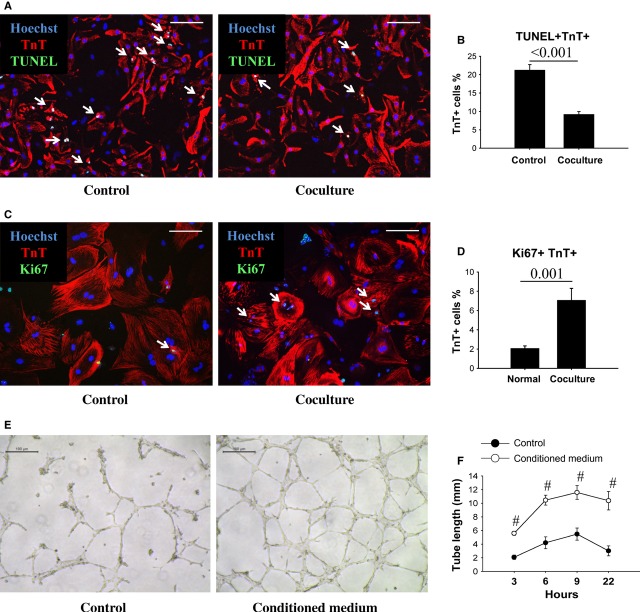
The cytokines from EnSCs enhanced cell survival, proliferation and tube formation *in vitro*. (**A**) Representative pictures of TUNEL-positive cardiomyocytes (white arrows). Scale bar denotes 100 μm. (**B**) Quantification of the apoptotic cardiomyocytes (*n* = 8/group). Coculture with EnSCs decreased the ratio of apoptotic cardiomyocytes as compared with control group. P values are shown at the top of bars. (**C**) Representative pictures of Ki67-positive cardiomyocytes (white arrows). Scale bar denotes 100 μm. (**D**) Quantification of proliferating cardiomyocytes *in vitro* (*n* = 9/group). Coculture with EnSCs increased the number of proliferating cardiomyocytes as compared with control group. P values are shown at the top of bars. (**E**) Representative pictures showed tube formation of HUVECs. Scale bar denotes 100 μm. (**F**) Quantitative analysis of the tube length (*n* = 4/group at each time-point). The EnSCs conditioned medium increased tube length as compared with control group. #*P* < 0.01 *versus* control group.

Next, we cocultured EnSCs with cardiomyocytes in a transwell system to study possible paracrine effects of EnSCs on cardiomyocytes. After simulated ischaemia for 48 hrs, coculture with EnSCs resulted in ∼50% fewer TUNEL-positive cardiomyocytes relative to culture alone (Fig. 4A and B). To evaluate a possible paracrine effect of EnSCs on cardiomyocyte proliferation, the coculture period was extended for an additional 8 days. Proliferating cardiomyocytes were detected by double immunostaining of Ki67 and TnT (Fig. 4C). Coculture increased the dual Ki67/TnT-positive cells by about threefold relative to the control group (Fig. 4D). Conditioned medium from EnSC cultures also enhanced tube formation of HUVECs (Fig. 4E). HUVEC tube length was significantly increased in the presence of conditioned medium at all time-points (Fig. 4F). These results confirmed that cytokines released from EnSCs are cardioprotective, possibly mitogenic and pro-angiogenic, and may stimulate cardiac myocyte re-entry into the cell cycle.

### Paracrine effect of EnSCs reduced cell apoptosis *in vivo*

**Fig. 5:**
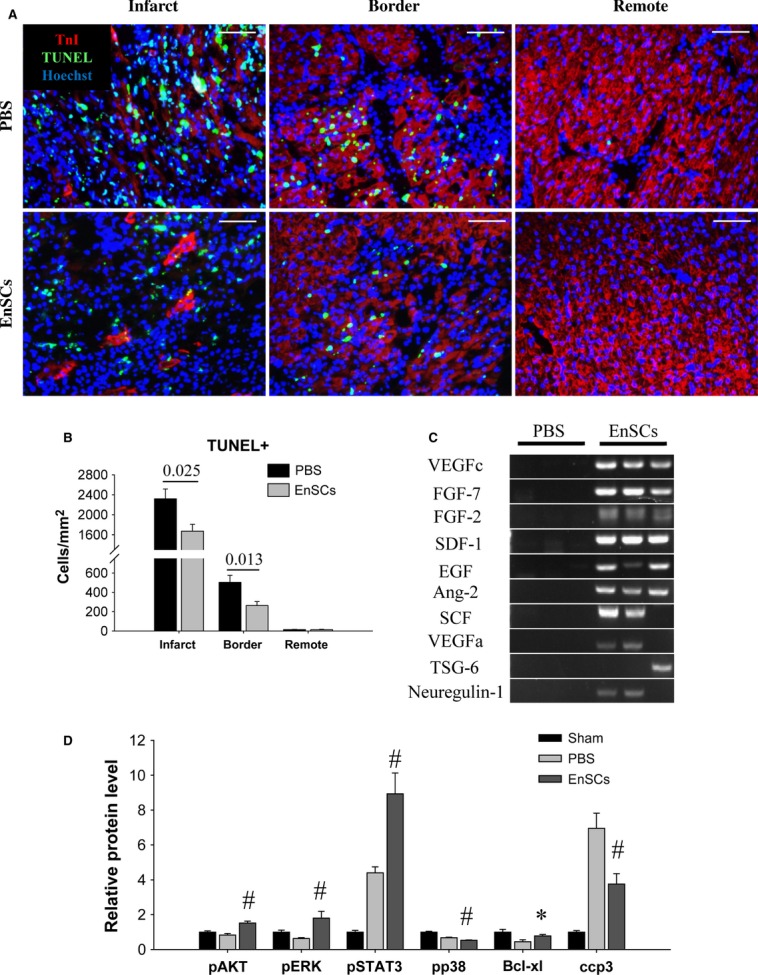
Transplantation of EnSCs reduced cell apoptosis *in vivo*. Rats transplanted with EnSCs were killed at 2 days. The hearts were collected for analysis. (**A**) Representative pictures of TUNEL-positive nuclei. Scale bar denotes 50 μm. (**B**) Quantification of the apoptotic nuclei (*n* = 5/group). EnSCs transplantation reduced apoptotic nuclei density in both infarct and border zone. P values are shown at the top of bars. (**C**) Representative picture of DNA products of PCR. Human-specific cytokine gene expression was detected from the hearts of EnSCs group. (**D**) Quantification of proteins detected by western blot (*n* = 3–5/group). Transplantation of EnSCs increased phosphorylation of AKT, ERK and STAT3, increased Bcl-xl expression and inhibited caspase3 cleavage. *P < 0.05 *versus* PBS group; #*P* < 0.01 *versus* PBS group.

We further validated the myocardial protective effect of EnSCs in vivo by TUNEL staining of LV sections (Fig. 5A). Transplantation of EnSCs markedly decreased apoptotic nuclei in both the infarct and border zone as compared with the PBS group at 2 days (Fig. 5B). The expression of human-specific cytokines was detected by RT-PCR at 2 days (Fig. 5C), and confirmed the expression of multiple protective cytokines by EnSCs *in vivo*.

To explore the possible mechanism for the paracrine-protective effect, we measured the phosphorylation of survival kinases in extracts of heart tissue (Figure S3). The phosphorylation of AKT, ERK and STAT3 increased, whereas that of p38 decreased in the EnSC group as compared with the PBS group, indicating selective activation of these pro-survival pathways by EnSCs transplantation at 2 days. Transplantation of EnSCs also resulted in enhanced expression of Bcl-xl and inhibition of caspase-3 cleavage (Fig. 5D).

### Paracrine stimulation of endogenous regeneration by EnSCs

**Fig. 6:**
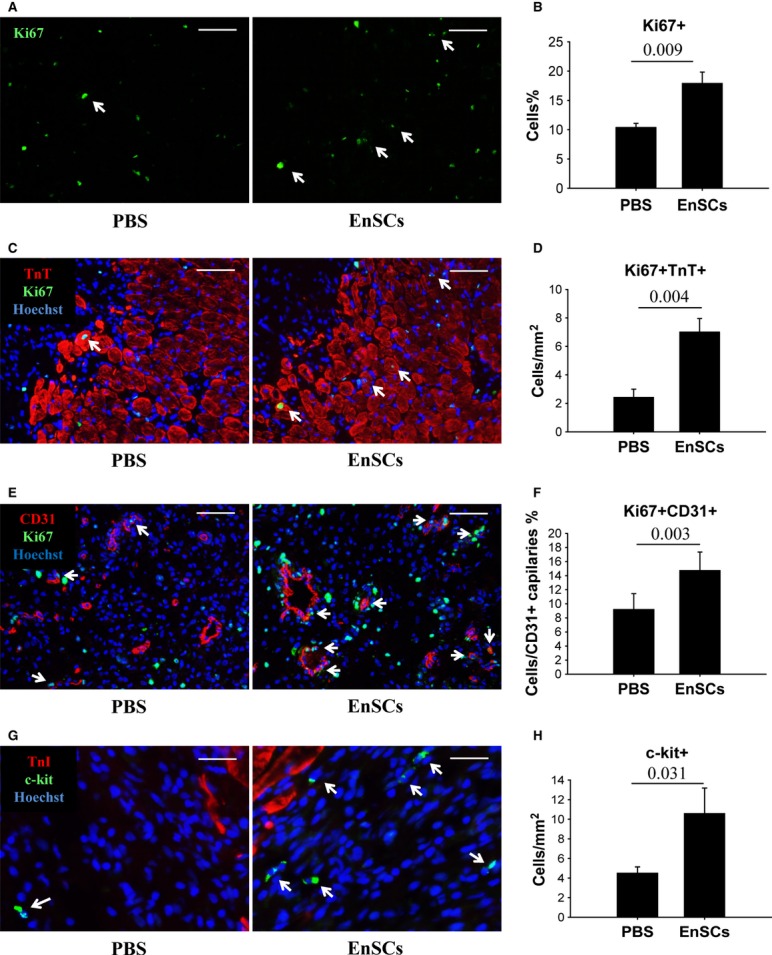
Transplantation of EnSCs promoted cell proliferation and c-kit-positive cell recruitment *in vivo*. Rats transplanted with EnSCs were killed at 7 days. The hearts were collected for immunofluorescent staining. (**A**) Representative pictures of immunostaining of Ki67+ cells in the infarct border (white arrows indicated proliferating cardiomyocytes). Scale bar denotes 100 μm. (**B**) Quantification of Ki67+ cells. (**C**) Representative pictures of colocalizing Ki67+ with TnT+ cells to demonstrate the proliferating cardiomyocytes (white arrows). Scale bar denotes 100 μm. (**D**) Quantification of Ki67+ TnT+ cells (*n* = 5–6/group). (**E**) Representative pictures of colocalizing Ki67+ (green) with CD31+ cells (red) to demonstrate the proliferating endothelial cells (white arrows). Scale bar denotes 50 μm. (**F**) Quantification of Ki67+ CD31+ cells (*n* = 5–6/group). (**G**) Representative pictures of c-kit+ cells (white arrows). Scale bar denotes 25 μm. (**H**) Quantification of c-kit+ cells (*n* = 8–9/group). P values are shown at the top of bars.

Increased viable myocardium within the infarct zone seen during 7–28 days after ligation in the EnSC group may involve endogenous myocardial regeneration. To examine the possible regenerative process, immunostaining of Ki67 was performed to assess proliferative cells in the infarct border zone at 7 days (Fig. 6A). The overall number of proliferating cells was increased by about 60% by EnSC transplantation (Fig. 6B). Proliferating cells were further analysed by colocalizing Ki67 with TnT (Fig. 6C) or the endothelial cell marker CD31 (Fig. 6E). EnSC transplantation increased the apparent proliferation of both cardiomyocytes (Fig. 6D) and endothelial cells (Fig. 6F). Significant more Ki67+ cardiomyocytes were found around the EnSCs, suggesting a paracrine effect (Figure S4). Because host stem cell recruitment may contribute to endogenous regeneration, we also quantified c-kit-positive cells. As shown in Figure 6G, the number of c-kit-positive cells increased by twofold in the EnSC group as compared with PBS (10.56 ± 2.61/mm^2^ *versus* 4.49 ± 0.64/mm^2^, *P* = 0.031).

### EnSC transplantation increased vascular density *in vivo*

**Fig. 7:**
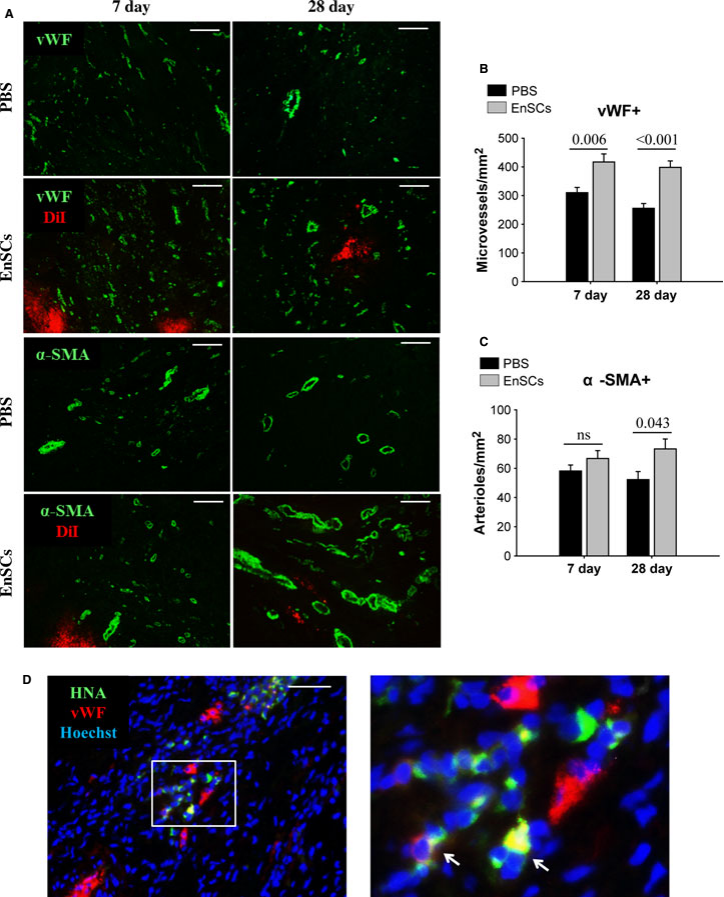
Transplantation of EnSCs stimulated angiogenesis *in vivo* through paracrine effect. (**A**) Representative immunofluorescence staining of vWF-positive microvessels and α-SMA-positive arterioles. Scale bar denotes 100 μm. (**B** and **C**) Quantification of vWF-positive microvessel and α-SMA-positive arteriole density (*n* = 5–6/group at each time-point). The vWF-positive microvessel density was greatly increased in EnSC group at 7 and 28 days. Arteriole density did not differ at 7 days, but was higher at 28 days in EnSC group than PBS group. P values are shown at the top of bars. (**D**) Representative image of human nuclear antigen–positive and vWF-positive cells at 28 days (white arrows). Scale bar denotes 50 μm.

Microvessels and arterioles were determined by immunostaining of vWF and α-SMA respectively (Fig. 7A). Microvessel density in the EnSC-treated hearts was significantly higher than that of the PBS group at both 7 and 28 days (Fig. 7B). EnSCs transplantation also resulted in a higher arteriole density at 28 days (Fig. 7C). We also assessed evidence for direct differentiation of EnSCs into endothelial cells and smooth muscle cells by dual antibody staining. Low numbers of vWF-positive EnSCs were detected, suggesting the possibility of low-level endothelial-like differentiation. However, positive cells were not integrated into the vasculature (Fig. 7D). No α-SMA-positive EnSCs were observed at 28 days. These data suggest that differentiation of EnSCs into vascular cells occurs at a negligible level and is unlikely to contribute to vascular/myocardial regeneration.

## Discussion

Our results support previous work demonstrating that cell therapy by direct intramyocardial delivery of MSCs exerts cardioprotection [16, 17] and supports tissue regeneration [18], thereby improving myocardial function and reducing adverse post-infarct remodelling. Our results further indicate that paracrine effects of human EnSCs are the primary and perhaps exclusive mechanism of tissue salvage and regeneration. In addition, we show that MSCs from menstrual blood secrete a distinctive set of cytokines and growth factors that are different from bone marrow–derived MSCs.

It was recently reported that cells of uterine and probably endometrial origin might contribute to myocardial regeneration and protection in healthy pre-menopausal female rats by migration into the infarcted myocardium [19]. The authors indicated that neither CD34+ nor Sca-1+ cell populations were implicated, nor the actual reparative cell type was defined. In our studies, we found that purified EnSCs conferred similar degrees of cardioprotective, revascularization and cardiac regeneration in male rats. Therefore, we propose that EnSCs may be the reparative cell type in the phenotypes described by Xaymardan *et al*. [19]. In addition to angiogenesis and improved functional outcome, we confirmed an increased area of viable myocardium in the infarct zone mediated by EnSC transplantation during 7–28 days. Histological analysis indicated no evidence of cardiomyocyte hypertrophy (Figure S5) and low differentiation of EnSCs into cardiomyocytes, smooth muscle or endothelial cells. However, we found increased numbers of c-kit+ cells in the infarct border of hearts in the EnSC group as compared with PBS and a highly significant, threefold parallel increase in Ki67-positive cardiomyocytes. We conclude that EnSCs can activate host cells and mediate the recruitment of c-kit-positive cells that contribute to regeneration of the injured myocardium [20]. In addition to its expression on cardiac stem cells, c-kit is also expressed on mast cells and other cell types of haematopoietic origin. Therefore, these cell types as well as cardiac stem cells could be present at accentuated levels during infarction and enhanced by EnSC therapy. The early apparent functional benefit of EnSC therapy at 7 days was not complemented by further improvement at 28 days (deterioration during 7–28 days was similar in both groups). Based on echocardiography, it is possible that scar tissue hampers the contraction of myocardium, so that the benefits of cell therapy are masked. If this is the case, it may be beneficial to combine stem cell transplant with medications that reduce collagen deposition [21].

Despite poor survival and differentiation, transplanted EnSCs conferred both myocardial protection and regeneration. The former is most likely mediated by survival kinase pathways activated in response to EnSC-secreted cytokines and growth factors that prevent necrosis and programmed death. Transactivation of these survival/proliferation kinase pathways may also be instrumental in the promotion of cell proliferation [22]. Therefore, we propose that tissue salvage, including a component of endogenous regeneration as well as cardioprotection, is mainly and perhaps exclusively the result of paracrine activity. We acknowledge that the TUNEL assay used does not discriminate the cell types undergoing apoptosis *in vivo*; however, MI is well known to include cardiac myocytes as well as other cell types and we interpret the results to indicate a cross-section of these cells. These results confirm a cytoprotective, anti-apoptotic property of EnSC therapy and are consistent with the protective paracrine activity that was demonstrated *in vitro* (Fig. 5A).

A major finding of our study is a 15% improvement of EF achieved by EnSCs transplantation at both 7 and 28 days after MI. This result suggests that early cardioprotection through paracrine effects of allogeneic MSCs is a viable strategy for the treatment of MI. The paracrine nature of protection also suggests that the secreted cytokine profile will be of paramount importance for clinical application. To this end, it will also be critical to establish lines of MSCs with optimal secretomes. In our studies, we compared cytokine production by EnSCs with that of BMMSCs under different culture condition. VEGF is considered to be a critical paracrine factor for BMMSCs-mediated cardioprotection [23]. VEGF expression was high in BMMSCs, but was undetected in EnSCs in the absence of hypoxia; conversely, EGF and TGFβ2 were expressed several orders of magnitude higher in EnSCs relative to BMMSCs, while periostin and PDGF levels were significantly elevated in EnSCs cultured under hypoxic conditions. These factors promote cellular proliferation, differentiation and survival [24–26].

The question arises whether optimally designed cell-free cytokine-based therapy may be preferable or even superior to live cell-based therapy. From this perspective, it can be noted that single cytokine therapy has failed in clinical trials [27, 28], whereas stem cell therapy is still commanding global attention [29]. It is likely that the combination of multiple secreted cytokines with different mechanisms of action and a more sustained and possibly conditional production capacity mediate the powerful paracrine effect of cell therapy that may be preferable to a cell-free system. Another reason why cell-free cytokine therapy may be elusive involves technical difficulties in identifying the optimal secretome [11] as well as the expense of large-scale production of cytokines [30]. It seems likely that optimized cell therapy will be the focus of continued basic and clinical research until improved technology is developed to facilitate cytokine therapy.

Our conclusion that EnSC therapy works primarily through paracrine mechanisms rather than differentiation is in contrast, although not incompatible with the implications of Hida *et al*. [31]. The conclusion has important implications for stem cell therapy. Tissue loss during MI is rapid whether the heart is reperfused. Therefore, in the absence of major regeneration and assuming a primary cardioprotective effect, it is essential that cell therapy be implemented as early as possible after ischaemia to prevent infarct expansion [32]. Such early treatment will only be possible by an ‘off the shelf’ strategy of cell therapy such as is possible using allogeneic cells. Ischaemia creates a harsh microenvironment not conducive for cell engraftment; however, it is possible that the death of newly transplanted cells actually enhances the paracrine effect by releasing intracellular contents [33]. In our study, EnSCs were delivered 30 min. after coronary ligation and this may account for the observed cardioprotection mediated by rapid activation of AKT, ERK, STAT3 and inhibition of p38 [22, 34–36]. These effects mimic and may be superior to ischaemic preconditioning that can salvage up to 50% of the myocardium during acute AMI in animal models [37].

All major stem cell clinical trials for AMI and ischaemic cardiomyopathy over the past decade have used autologous cells [38, 39]. At the present time, these cells must be harvested and cultured from each patient and this may preclude early intervention and also result in therapeutic variation caused by patient-related differences in the quality of the cells [40, 41]. This time-related limitation may not apply to cardiac stem/progenitor cells because these cells have documented regenerative and infarct-healing potential even at late delivery times [38]. EnSCs provide obvious practical benefits for ‘off the shelf’ regenerative medicine to circumvent such limitations, especially related to MSC therapy, because of their availability, easy access and culture, and embryonic-like quality [7, 31]. A major risk for allogeneic cell therapy is immunorejection, but this is minimized by MSCs that are immunoprivileged. In our study, we purposely used non-immunosuppressed xenotransplantation as our model and showed efficacy of therapy even on this background. This may be of significant clinical relevance. Although human EnSCs were cleared from the rat heart within the 28-day experimental period, presumably because of removal by macrophages (Figure S6), a significant population was retained at 7 days when the ‘apparent’ myocardial regeneration and angiogenesis effects were most significant. No apparent collateral damage appeared to result from the adverse immunoreaction and the functional benefits were clear and significant. It is possible that the immune modulatory properties of EnSCs neutralize inflammation in a secondary reaction during MI [42]. We cannot dismiss the possibility that improved cell survival would increase therapeutic benefit. In clinical trials for MI, allogeneic BMMSCs have been reported to have similar safety and efficacy as autologous BMMSCs [43–45]. Similarly, when patients with multiple sclerosis received intravenous injection of allogeneic EnSCs, there was no immediate immune reaction or tumour formation [46]. Finally, it may be possible to establish an EnSC bank that contains a range of HLA-typed cell lines that could minimize the risk of immunorejection and help overcome the immunological barrier.

In conclusion, we demonstrate that xenogeneic transplantation of EnSCs preserves myocardium, stimulates endogenous regeneration and improves heart function in a rat MI model. EnSCs may have superior paracrine properties compared with other cell types including BMMSCs, and we conclude from our analysis that the therapeutic effect of EnSCs occurs principally and perhaps exclusively through paracrine effects. The indirect mechanism is consistent with the observed functional benefits that occur despite progressive cell clearance. This study supports further investigations into EnSCs as potential ‘off the shelf’ clinical reagents for cardiovascular cell therapy.

## Supporting information

**Figure S1.** Study design.

**Figure S2.** Comparison of *in vitro* cytokine expression of EnSCs and BMMSCs under normoxia and hypoxia/serum deprivation condition.

**Figure S3.** EnSCs activated survival pathways *in vivo*.

**Figure S4.** Proliferating cardiomyocytes were found around the EnSCs at 7 days.

**Figure S5.** Cardiomyocyte size and morphology.

**Figure S6.** EnSCs were cleared by macrophage *in vivo*.
