# Supplementary figures and images for: Human endometrial stem cells confer enhanced myocardial salvage and regeneration by paracrine mechanisms
Source: J Cell Mol Med. 2013 Jul 9;17(10):1247–60. doi: 10.1111/jcmm.12100 (PMC3843975; doi:10.1111/jcmm.12100)

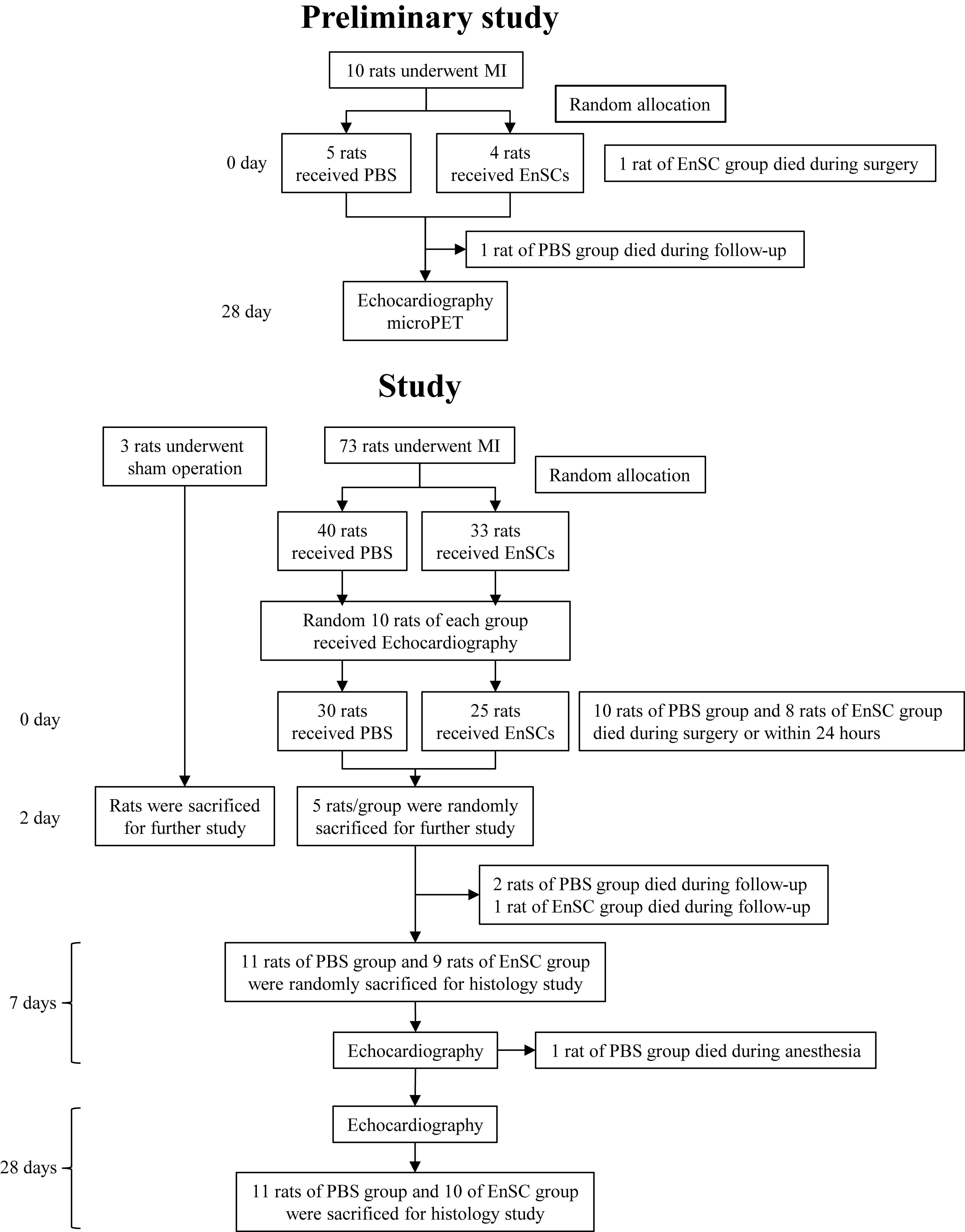

Supplement: Supplementary file 1 [file jcmm0017-1247-SD1.tif]

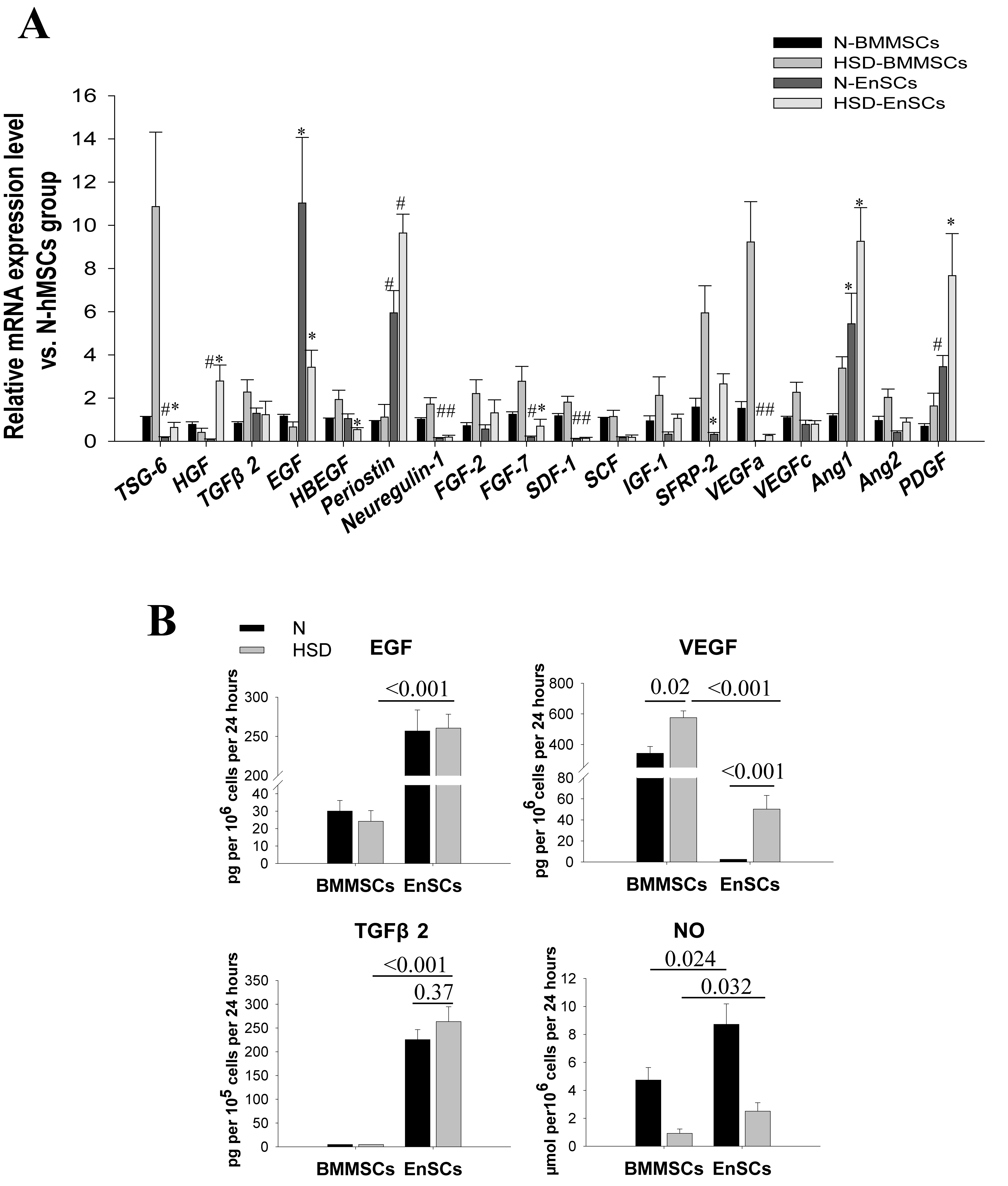

Supplement: Supplementary file 2 [file jcmm0017-1247-SD2.tif]

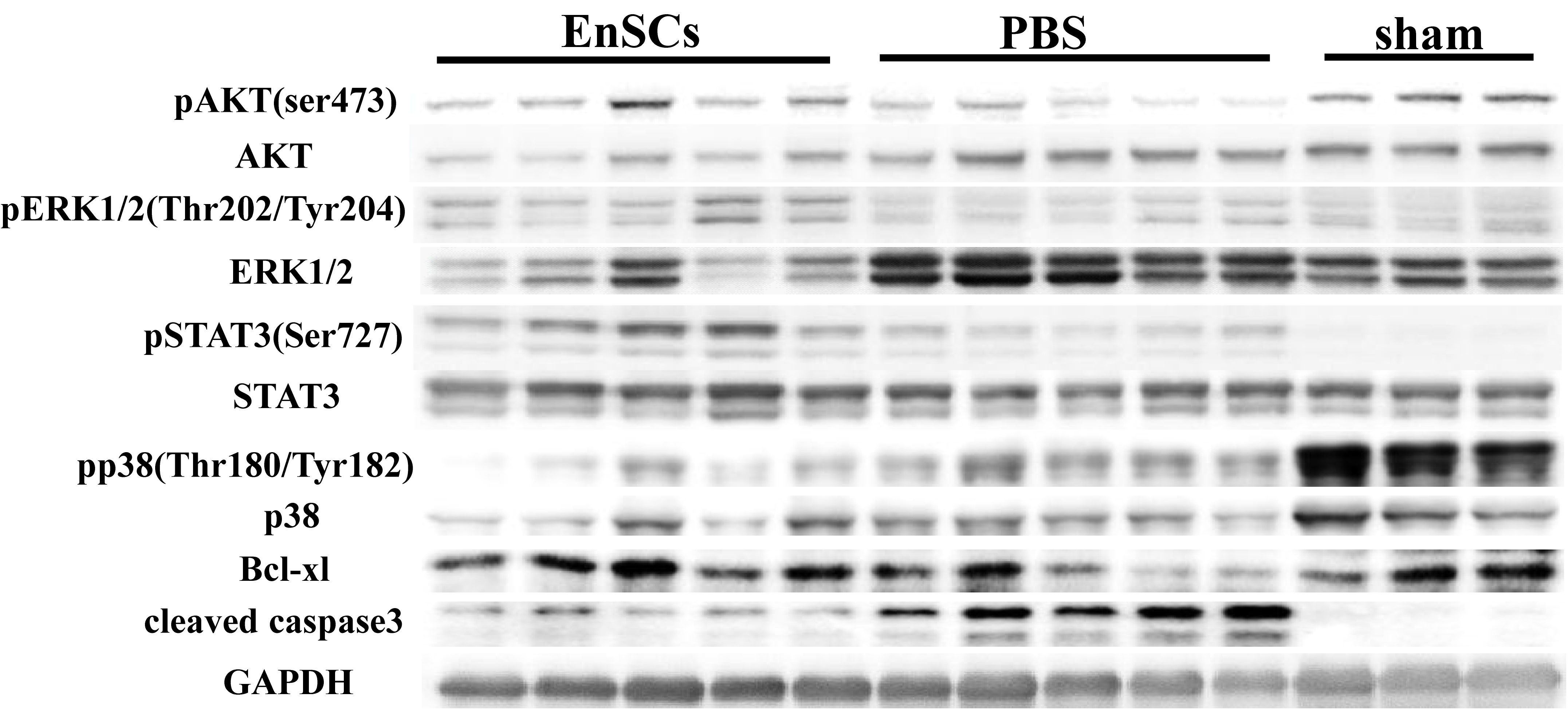

Supplement: Supplementary file 3 [file jcmm0017-1247-SD3.tif]

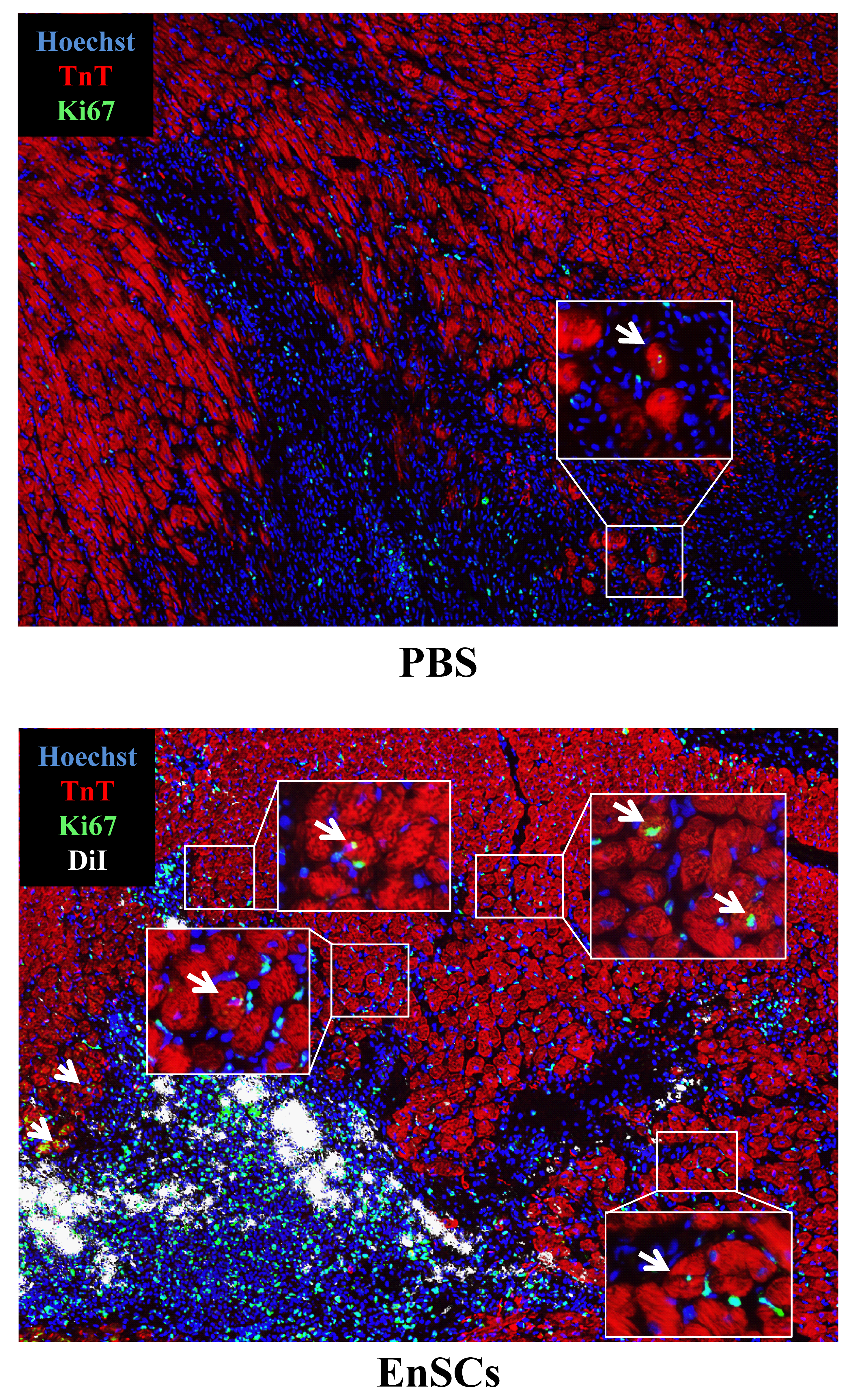

Supplement: Supplementary file 4 [file jcmm0017-1247-SD4.tif]

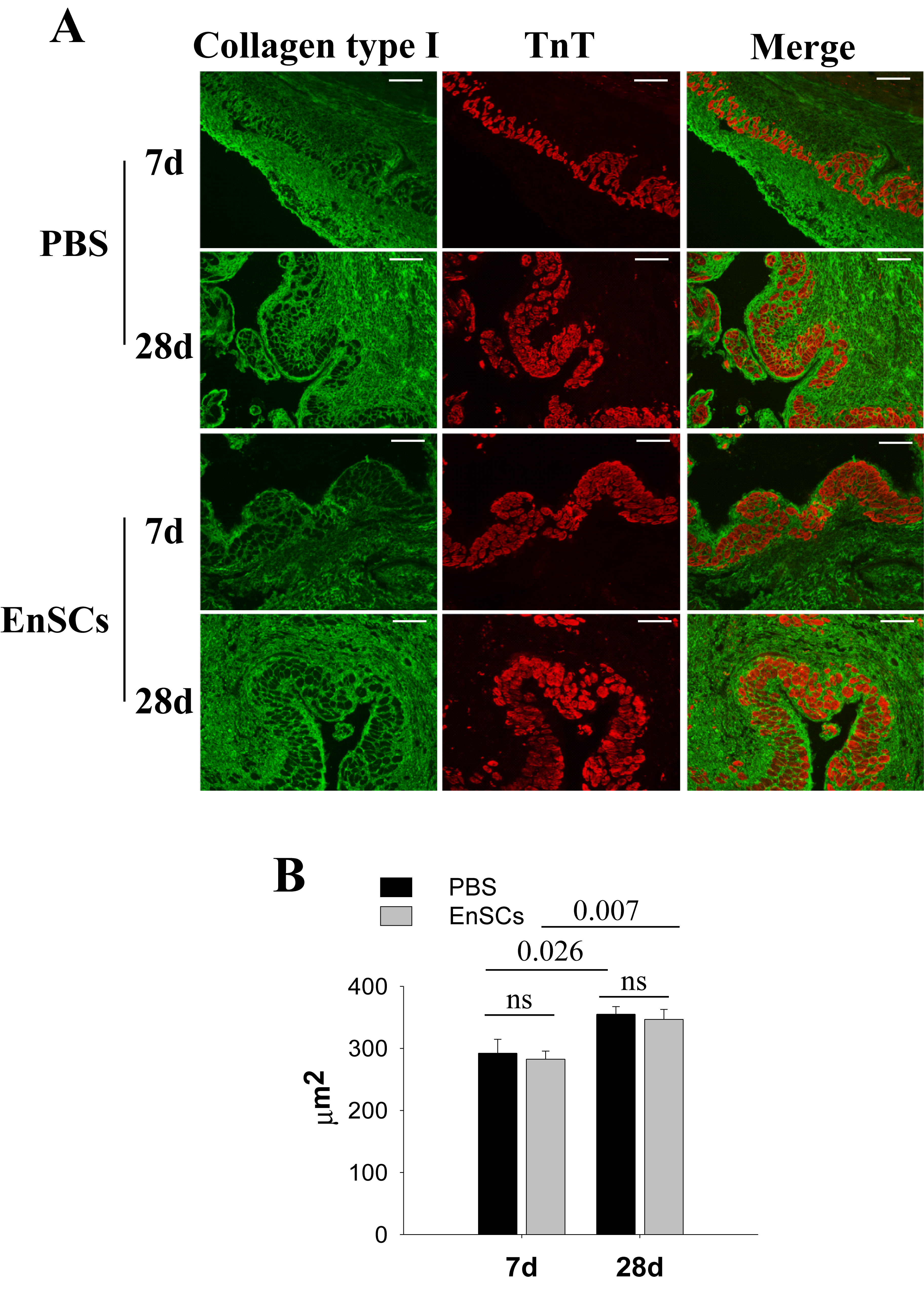

Supplement: Supplementary file 5 [file jcmm0017-1247-SD5.tif]

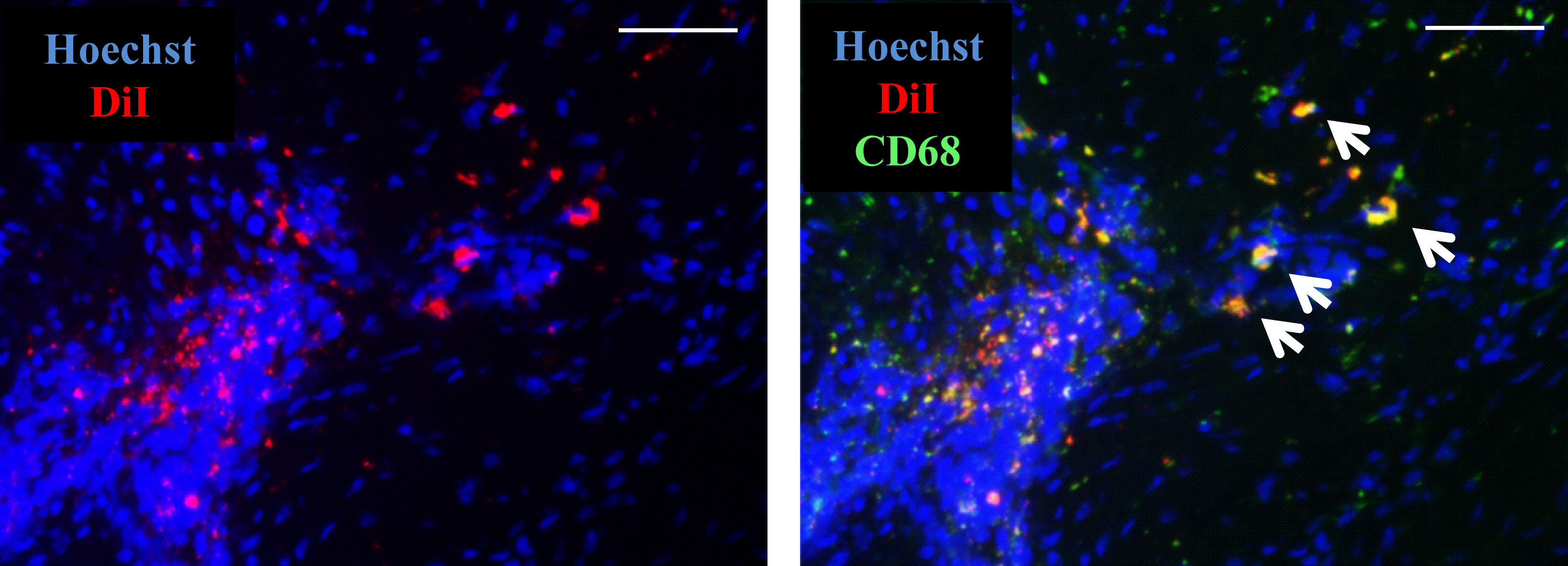

Supplement: Supplementary file 6 [file jcmm0017-1247-SD6.tif]
