# Supplementary material for: Human endometrial stem cells confer enhanced myocardial salvage and regeneration by paracrine mechanisms
Source: J Cell Mol Med. 2013 Jul 9;17(10):1247–60. doi: 10.1111/jcmm.12100 (PMC3843975; doi:10.1111/jcmm.12100)
Supplement: Supplementary file 7 [file jcmm0017-1247-SD7.doc]

**SUPPLEMENTARY INFORMATION**

**Materials and Methods**

***EnSCs preparation***

EnSCs of passage 8-14 were used in all experiments. Before transplantation, EnSCs were labeled by incubation in serum free DMEM/F12 (Gibco, USA) containing 5μmol/l DiI (Invitrogen, USA) for 30min at 37℃. The cells were rinsed with PBS, detached with 0.25% (w/v) Trypsin EDTA (Gibco, USA) and neutralized in complete medium. Cells were centrifuged and washed 3X with PBS. After a final centrifugation, the cells were suspended in PBS at 1.5×10^6^ cells per 150 μl. 30 minutes after the coronary ligation cells were directly injected into the ischemic border zone at 5 sites. MI Control rats received the same volume of cell free PBS.

***Flow cytometric analysis***

EnSCs were suspended in 100ul PBS at 5×10^5^ per tube and incubated with primary antibodies or isotype-matched control antibodies on ice for 1 hour. Then the cells were washed 3X with PBS and analyzed by flow cytometry (Becton Dickinson, CA). Antibodies against CD29, CD34, CD45, CD90, CD105, CD117, CD133 and CD166 were purchased from BD PharMingen and eBioscience.

***Cell proliferation and colony formation assay***

EnSCs of passage 9 and BMMSCs of passage 4 were seeded into four 96-well plates at the density of 1000 cells/well with 100ul of complete culture medium. After adhesion for 3, 24, 48 and 72 hours, the supernatant was removed and 100ul complete culture medium containing 10ul of CCK8 was added to each well for another 3 hours at 37℃. Then the plate was shaken for 10min and the optical density (OD) values were read at 450 nm. The OD value at 3 hours (OD^3h^) served as baseline, relative cell number at timepoint was calculated from OD^timepoint^ /OD^3h^.

EnSCs of passage 9 and BMMSCs of passage 4 were seeded into 6-well plates at the density of 200cells/cm^2^ with complete culture medium containing 10% FBS. The medium were changed every 2 days. After 14 days of incubation, the cells were fixed in 10% formalin for 5 minutes and washed twice with PBS. Then crystal violet staining was used to shown the colonies.

***Collection of conditioned medium***

EnSCs were grown to 90% confluence and the culture medium was replaced with 3ml of DMEM containing 2% fetal bovine serum. Cell-free medium served as control. After 24 hours of normoxic incubation, the conditioned medium was collected and centrifuged at 12000rpm for 10min. The supernatant was used immediately for assays.

***Tube formation assay***

200ul of Matrigel was added into wells of a 24-well plate and incubated at 37℃ for 30min. Immortalized human umbilical vein endothelial cells (HUVECs) were seeded into the Matrigel prepared wells (8 x 10^4^ cells per well) with EnSCs conditioned medium or the DMEM containing 2% fetal bovine serum. Images were acquired by phase contrast microscopy (Leica) at 3, 6, 9, 12 and 21 hours.

***ELISA***

8×10^5^ EnSCs or BMSCs were seeded into 25cm^2^ flasks with complete medium and incubated 12 hours to allow cell adherence. The cells were washed three times with PBS and then divided into two groups. For normoxia, 3ml DMEM containing 10% fetal bovine serum was incubated under 21% O_2_ 5% CO_2_. For hypoxia, plates were exposed to 0.5% O_2_ 5% CO_2_. 24 hours later, the medium was collected, centrifuged at 12000rpm for 10min and stored at -80℃. Concentrations of TGF-β2, EGF and VEGF was measured by Quantikine ELISA kit (R&D Systems), following manufacture’s instruction. The data was standardized to cell number and expressed as pg per 10^5^ cells.

***RT-PCR analysis***

Total ribonucleic acid was extracted with Trizol reagent (Invitrogen, USA). Reverse transcription was performed with oligo (dT)18 using M-MLV reverse transcriptase (TAKARA) in a total volume of 20ul reaction volume. Quantitative PCR used SYBR Premix Ex Taq system (TAKARA). GAPDH or beta-actin genes were used as the internal control for PCR normalization. Amplified products were separated by 2% agarose gel electrophoresis and visualized by GelDoc^TM^ XRS+ imaging System (Bio-Rad).

***Isolation of Neonatal Rat Ventricular Cardiomyocyte***

Primary cultures of neonatal rat ventricular cardiomyocytes were prepared by the method originally described by Simpson and Savion with minor modifications . Briefly, 2-day-old Spragure-Dawley rats were sacrificed with pentobarbital. Hearts were minced and dissociated with 0.1% trypsin (Gibco, USA). The dispersed cells were incubated with high glucose DMEM (glucose concentration, 4g/L) containing 10% fetal calf serum and 100 umol/L 5’-bromo-2’-deoxyuridine (Sigma, USA) in 25cm^2^ flasks for 90min at 37℃ in humidified air with 5% CO_2_. Unattached cells were collected and seeded into 24-well plates (2.5×10^5^ cells/well) or 6-well plates (1×10^6^ cells/well) (Corning, USA). The culture medium was changed 48h after seeding. After 72h of incubation, cardiomyocytes were about 70% confluence and were used for experiments.

***Cardiomyocytes and EnSCs coculture assay***

Coculture experiments were performed in 24-insert transwell systems (Corning, 3um pore size), which prevents direct cell contact but preserves cell interaction by soluble factors. EnSCs were seeded on the semi-permeable membranes of the 24-well insert (1.5×10^4^ per insert) and incubated 24 hours to allow cell adherence. The EnSCs inserts were placed into plates with cardiomyocytes. For proliferation assay, medium was changed every other day for 8 days. For apoptosis assay, medium was changed with serum free low glucose DMEM (glucose concentration, 1g/L) and followed by 48 hours of hypoxia (less than 0.1% O_2_) in an airtight Plexiglas chamber with 5% CO_2_ and 95% N_2_ at 37℃.

***Rat model of myocardial infarction***

A total of 86 male Sprague-Dawley rats (200-250 g) were subject to permanent ligation of the left anterior descending coronary artery to create MI as described previously . Briefly, rats were intubated under general anesthesia with intraperitoneal injection of pentobarbital (50mg/kg of body weight) and ventilated with room air by using a small animal ventilator (Zhejiang University Apparatus, China). MI was induced by permanent ligation of the left anterior descending coronary artery with a 6-0 silk suture. Successful performance of coronary occlusion was verified by the observation of blanching of the myocardium distal to the coronary ligation. The sham-operation group received thoracotomy without coronary ligation.

***Echocardiography***

Echocardiography was performed at baseline, 7 days and 28 days after surgery. Rats were anesthetized with pentobarbital (50mg/kg of body weight), placed in the supine position on a heating pad and their chests were shaved. Then trans-thoracic 2-dimensional and M-mode echocardiographic (17.5MHz transducer, Vevo 2100, VisualSonics, Canada) images at the papillary muscle level were obtained by a blinded investigator. All measurements were means of at least 3 continuous cardiac cycles. Left ventricular volumes were calculated from the Teichholtz formula: EDV=7.0/(2.4+LVIDd)×LVIDd^3^ and ESV=7.0/(2.4+LVIDs)×LVIDs^3^, where EDV and ESV are end-diastolic and end-systolic volumes; LVIDd is the left ventricular internal dimension in diastole (d) and LVIDs is that in systole (s). Ejection fraction (EF) was determined from (EDV-ESV)/EDV×100%. Fraction shortening (FS) was calculated as (LVIDd-LVIDs)/LVIDd×100%. Intraventricular septum (IVS) movement was determined from (IVSs-IVSd)/IVSd×100%, where IVSs and IVSd are the thickness of IVS in systole (s) and diastole (d), respectively.

***Positron Emission Tomography***

Rats were injected with 0.6mCi 18-FDG by tail vein. Thirty minutes later, rats were anesthetized and sustained with inhalation of 2% isoflurane and placed in a spread prone position on a dedicated holder for imaging. A 15-min static data acquisition was performed in three-dimensional mode using a micro-PET imaging system (R4, Concorde Microsystems, USA). Image reconstruction was performed with attenuation and decay correction by maximum a posteriori algorithm. The corrections for dead time, random scattering were also performed. Then, transaxial, coronal, and sagittal tomographic slices were obtained. Contiguous 4-7 transaxial sections which contained distinct infarcted regions of the left ventricle were used for semi quantitative evaluation. We used the region of interest (ROI) method to measure the standard up take volume (SUV) of infarct regions and normal regions . The SUV of infarct region was divided by that of normal region to evaluate the relative volume of viable myocardium in the infarcted regions.

***Histology***

Rats were sacrificed by i.v. pentobarbital overdose at 2 days, 7 days and 28 days after MI. The hearts were quickly harvested and embedded in optimal cutting temperature compound (Sakura Finetek USA Inc., CA). Frozen sections of left ventricular samples were cut at 7μm thickness and stored at -80℃. Differentiation of EnSCs into cardiomyocytes, endothelial cells and smooth muscle cells was identified by immunostaining with antibodies against human nuclear antigen (HNA), Troponin T (TnT), von Willebrand factor (vWF) and α-smooth muscle actin (α-SMA). Proliferating cardiomyocytes and endothelial cells were evaluated by immunostaining against Ki67, TnT and CD31. The recruitment of endogenous stem cells was assessed by immunostaining against c-kit. Vessel density in the infarct border zone was evaluated by immunostaining against vWF and α-SMA. DyLight conjugated antibodies (Abcam, USA) were used and Hoechst 33258 (Invitrogen, USA) was used to stain nuclei. For morphometric measurement, the pixel region of collagen type I and its endocardial and pericardial myocardium, excluding the intact myocardium at infarct border, was defined as infarct zone. The pixel region of TnT in the infarct zone was defined as viable myocardium. Myocardium fraction was determined from myocardial area/infarct area×100%. Infarct size was calculated by dividing the sum of the endocardial and epicardial lengths of the infarct zone by the sum of the total epicardial and endocardial circumferences of the left ventricular. The size of cardiomyocyte was assessed at transverse area in the infarct zone (100 cells per heart). All the measurement used Image-Pro Plus.

***Terminal Deoxynucleotidyl Transferase Biotin-dUPT Nick end Labeling (TUNEL) in heart sections***

A TUNEL staining kit (DeadEnd™ Fluorometric TUNEL system, Promega, USA) was used to visualize cell death in plates or heart sections. After 10-min fixing with 10% buffered formalin phosphate and pretreatment with 0.2% Triton X-100, plates or sections were incubated in an equilibration buffer as per manufacturer’s instructions. TdT enzyme and nucleotide mix were added and the samples incubated for 60 min at 37℃. Plates or sections were washed with 2
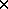
SSC washing buffer for 15 min followed by 3X PBS.

***Western blot analysis***

Tissue samples which were ground to powder in liquid nitrogen; cultured cells were lysed with modified RIPA buffer (50mM Tris, pH 8.0, 0.5% sodium deoxycholate, 1% Triton X-100, 0.1% SDS, 150 mM NaCl, 1 mM EDTA) containing proteinase inhibitors (Sigma) for 30min on ice, followed by centrifugation at 14,000g at 4℃ for 30 min. Protein concentration was determined by the BCA protein assay (Pierce). 20-40 μg of protein per sample was electrophoresed on 9 or 15% gels by SDS-PAGE and transferred to PVDF membranes (Bio-Rad). The membranes were blocked with 5% non-fat milk in PBS containing 0.1% Tween-20 (PBS-T) at room temperature for 1 hour, and incubated overnight at 4℃ with specific primary antibodies. After 3X washing with PBS-T, HRP-conjugated secondary antibodies were added for 1 hour at room temperature. Membranes were washed 3X with PBS-T, incubated with ECL (Millipore) for 1min and exposed in ChemiDoc^TM^ XRS+ imaging System (Bio-Rad). Densitometric analysis was carried out by Quantity One (Bio-Rad).

**References**

1. **Simpson P, Savion S.** Differentiation of rat myocytes in single cell cultures with and without proliferating nonmyocardial cells. Cross-striations, ultrastructure, and chronotropic response to isoproterenol. *Circ Res*. 1982; 50: 101-16.

2. **Hu X, Yu SP, Fraser JL, et al.** Transplantation of hypoxia-preconditioned mesenchymal stem cells improves infarcted heart function via enhanced survival of implanted cells and angiogenesis. *J Thorac Cardiovasc Surg*. 2008; 135: 799-808.

3. **Morooka M, Kubota K, Kadowaki H, et al.** 11C-Methionine PET of Acute Myocardial Infarction. *J Nucl Med*. 2009; 50: 1283-7.

**Supplemental figure legends**

**Supplemental Figure 1. Study design.** Overall mortality due to surgery was 22.89% within 24h with no difference between groups. In total 4 rats from the PBS group and 1 from the EnSC group died during follow-up.

**Supplemental Figure 2. Comparison of in vitro cytokine expression of EnSCs and BMMSCs under normoxia and hypoxia/serum deprivation condition.** A. Relative mRNA level determined by real-time PCR (n=3/group). EGF, periostin, Ang1 and PDGF expression were significantly higher in EnSCs than BMMSCs. *p<0.05 versus BMMSCs of the same culture condition, #p<0.01 versus BMMSCs of the same culture condition. B. Protein level of cytokines determined by ELISA (n=3/group). BMMSCs and EnSCs showed different secretome. p values were shown at the top of bars. Abbreviations: N, normal culture; HSD, hypoxia and serum deprivation.

**Supplemental Figure 3. EnSCs activated survival pathways in vivo.** Rats transplanted with EnSCs were sacrificed at 2 days. Representative western blots showed protein phosphorylation and expression among groups.

**Supplemental Figure 4. Proliferating cardiomyocytes were found around the EnSCs at 7 days.** White arrows indicated Ki67 positive cardiomyocytes.

**Supplementary Figure 5. Cardiomyocyte size and morphology.** Rats transplanted with EnSCs were sacrificed at 7 and 28 days and myocardial sections analyzed by histology. A. Representative image of collagen type I and TnT displaying cardiomyocyte size in the infarct region at different time points. Scale bar denotes 100μm. (B) Quantitative analysis of cardiomyocyte area (n=9 for each timepoint). Area was measured in fields of perpendicular sectioned cardiomyocytes. p values are shown at the top of bars.

**Supplemental Figure 6. EnSCs were cleared by macrophage in vivo.** Rats transplanted with DiI labeled EnSCs were sacrificed at 7 and 28 days. The hearts were cryosectioned for histological analysis. CD68+ macrophages are positive for DiI (white arrows), indicating EnSC phagocytosis by macrophages. Scale bar denotes 50μm.
